# Phytoene Desaturase from *Oryza sativa*: Oligomeric Assembly, Membrane Association and Preliminary 3D-Analysis

**DOI:** 10.1371/journal.pone.0131717

**Published:** 2015-07-06

**Authors:** Sandra Gemmecker, Patrick Schaub, Julian Koschmieder, Anton Brausemann, Friedel Drepper, Marta Rodriguez-Franco, Sandro Ghisla, Bettina Warscheid, Oliver Einsle, Peter Beyer

**Affiliations:** 1 Faculty of Biology, Cell Biology, University of Freiburg, Freiburg, Germany; 2 Faculty of Biology, Biochemistry and Functional Proteomics, University of Freiburg, Freiburg, Germany; 3 Faculty of Chemistry and Pharmacy, Institute for Biochemistry, University of Freiburg, Freiburg, Germany; 4 Department of Biology, University of Konstanz, Konstanz, Germany; 5 BIOSS Centre for Biological Signalling Studies, University of Freiburg, Freiburg, Germany; University of Manitoba, CANADA

## Abstract

Recombinant phytoene desaturase (PDS-His_6_) from rice was purified to near-homogeneity and shown to be enzymatically active in a biphasic, liposome-based assay system. The protein contains FAD as the sole protein-bound redox-cofactor. Benzoquinones, not replaceable by molecular oxygen, serve as a final electron acceptor defining PDS as a 15-*cis*-phytoene (donor):plastoquinone oxidoreductase. The herbicidal PDS-inhibitor norflurazon is capable of arresting the reaction by stabilizing the intermediary FAD_red_, while an excess of the quinone acceptor relieves this blockage, indicating competition. The enzyme requires its homo-oligomeric association for activity. The sum of data collected through gel permeation chromatography, non-denaturing polyacrylamide electrophoresis, chemical cross-linking, mass spectrometry and electron microscopy techniques indicate that the high-order oligomers formed in solution are the basis for an active preparation. Of these, a tetramer consisting of dimers represents the active unit. This is corroborated by our preliminary X-ray structural analysis that also revealed similarities of the protein fold with the sequence-inhomologous bacterial phytoene desaturase CRTI and other oxidoreductases of the GR2-family of flavoproteins. This points to an evolutionary relatedness of CRTI and PDS yielding different carotene desaturation sequences based on homologous protein folds.

## Introduction

Carotenoids are yellow-orange pigments with various functions in plants ranging, *inter alia*, from coloration, through photosynthesis to phytohormone supply (abscisic acid and strigolactones). The chromophore of cyclic plant carotenoids consists of a polyene structure mostly comprising 11 conjugated double bonds. These double bonds are introduced by carotene desaturases, which come in two sequence-inhomologous classes and belong to either the PDS- or the CRTI clade.

**Fig 1:**
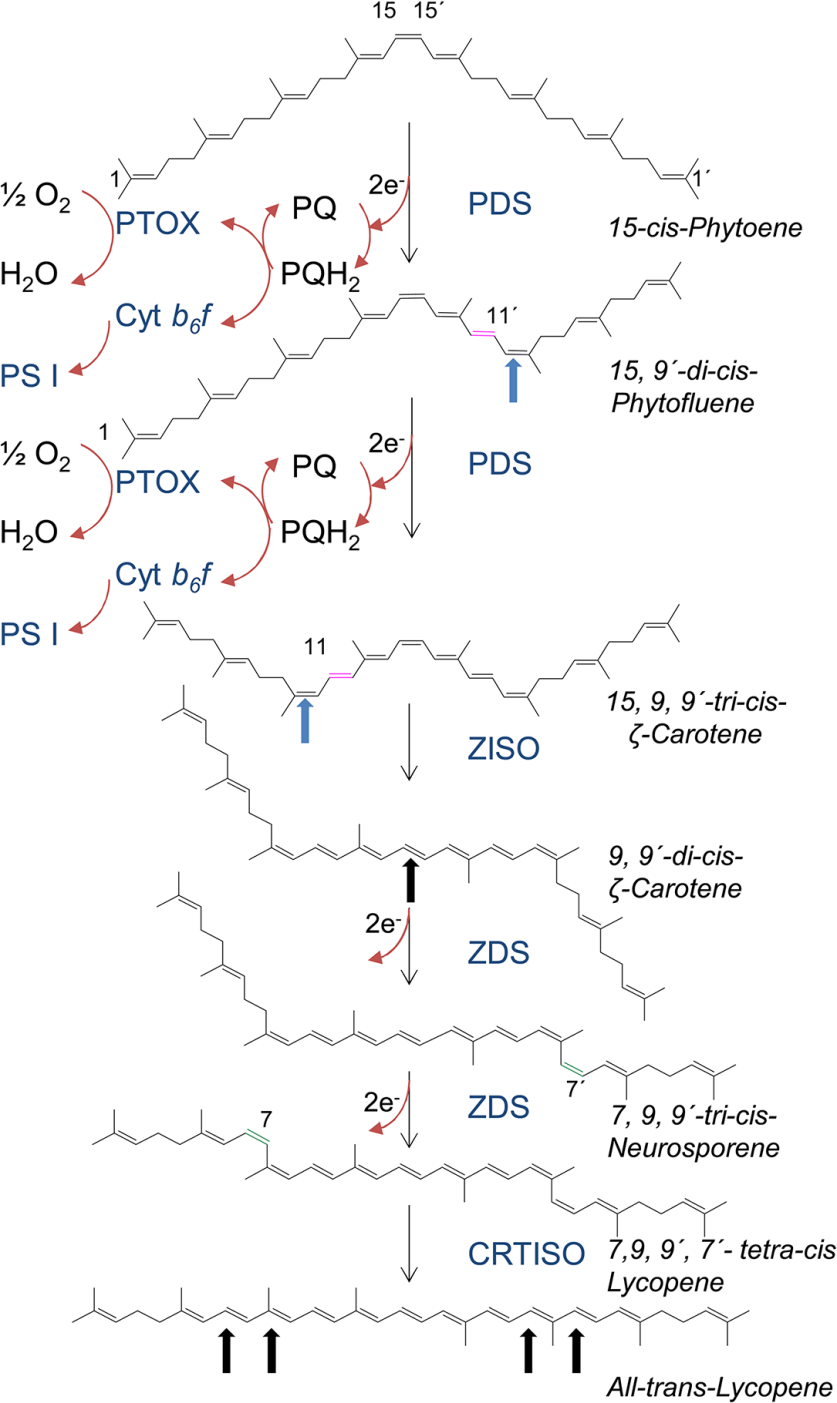
The PDS/ZDS carotene desaturation sequence. Magenta double bonds; introduced in *trans* configuration, green double bonds introduced in *cis* configuration. Blue arrows show isomerization from *trans* to *cis*, black arrows from *cis* to *trans*. Redox pathways in chloroplasts employ photosynthetic electron transport while a route through PTOX takes place in developing chloroplasts and non-green plastids. Both routes may as well prevail with ZDS. For further explanations, see text.

Phytoene desaturases (PDS), the subject of this work, prevail in plants and cyanobacteria; they introduce two double bonds into the symmetric, colorless phytoene substrate. This extends the triene chromophore of phytoene to form—via the pentaene intermediate phytofluene—the light yellow ζ-carotene with seven conjugated double bonds (Fig 1). The new double bonds are inserted symmetrically at positions C11 and C11´. A second, homologous desaturase, ζ-carotene desaturase (ZDS) is required to insert two additional double bonds at positions C7 and C7´. This leads to the red-colored lycopene with 11 conjugated double bonds.

PDS, a membrane-bound plastid-localized protein is known to be notoriously difficult to deal with. Homogeneous preparations of PDS have been obtained which, however, required complex additives such as fungal extracts [1] or plastid stroma [2] for activity. This has hampered investigations of its properties. Consequently, all mechanistic knowledge accumulated to date appears, in retrospect, as a conglomerate of conclusions drawn from data that stem from most disparate experimental approaches. Radiotracer experiments using crude chromoplast preparations [3] and PDS expressed in *E*. *coli* [4] revealed the presence of a complex poly-*cis* pathway that includes dehydrogenation and isomerization reactions. PDS introduces *trans* double bonds at C11 and C11´ of 15-*cis*-phytoene, thereby also converting the adjacent C9 and C9´double bonds from *trans* to *cis*. In contrast, ZDS introduces double bonds at the C7 and C7´position of ζ-carotene in *cis* configuration resulting in a C7,9,9´,7´-*tetra*-*cis*-lycopene species, termed prolycopene. Such poly-*cis* carotene intermediates accumulate in the *Tangerine* mutation of tomato fruit [5] that lacks the activity of the enzyme carotene *cis*-*trans* isomerase (CRTISO) [6–9]. One additional isomerase involved came to light through the identification of ζ-carotene *cis*-*trans* isomerase (ZISO) [10] that acts on the C15-C15´ *cis* double bond of the PDS product 9,15,9’-*tri*-*cis*-ζ-carotene. In chloroplasts, this enzymatic reaction can be replaced by photoisomerization apparently requiring a photosensitizer that is absent in non-green plastids [6]. The isomerization of this central double bond removes a blockage and is decisive for allowing ZDS catalysis [3]. Thus, two desaturases and two *cis*-*trans* isomerases are involved in the biosynthesis of all-*trans* lycopene, the molecule, which then undergoes end-cyclizations to form the α- and β-ionone functionalities present in the downstream carotenoids [11].

To date, there is no clear rationale for the purpose of such complexity in view of the fact that bacterial CRTI-type desaturases form all-*trans* lycopene directly from 15-*cis*-phytoene, introducing all four double bonds with all-*trans* stereochemistry [12]. However, recent evidence suggests that poly-*cis* carotene intermediates can be a starting point for the biosynthesis of molecules involved in feedback regulatory phenomena [13] and leaf development [14]. This seemingly adds to the notion that *cis* carotenoids sometimes constitute a “tag” for regulatory derivative formation such as strigolactones (from 9-*cis*-β-carotene [15] and abscisic acids (from 9-*cis*-violaxanthin [16].

A further issue pertains to the nature of the electron acceptor for PDS. The use of complex chromoplast systems indicated that quinones can serve as intermediate electron carriers [17], while oxygen acts as a terminal acceptor [3]. The role of quinones was corroborated by the finding that *Arabidopsis* mutants defective in plastoquinone biosynthesis were unable to desaturate phytoene [18]. Thus PDS, directly or indirectly, employs plastoquinone and then oxygen and maybe additional components of a more or less extended redox chain [19], an additional component of which was discovered through the IMMUTANS mutation of *Arabidopsis*. Phenotypically, this mutation is characterized by severe impairment of carotene desaturation caused by a dysfunctional plastid terminal oxidase (PTOX) [20], a highly specific plastoquinol:oxygen oxidoreductase [21]. The role of PTOX in carotene desaturation is crucial in non-green plastids, such as tomato fruit chromoplasts [22], while it is dispensable for carotene desaturation in chloroplasts. Here, the redox state of the plastoquinone pool is being dominated by the activity of the photosynthetic electron transport with which PTOX cannot compete [23]. The role of PTOX in chloroplasts is rather thought to be in the regulation of the balance between linear and cyclic electron transport. A function as a “safety valve” protecting thylakoids from over-reduction–in analogy with the related mitochondrial alternative oxidase–is controversially discussed [24, 25].

Current knowledge on PDS is largely based on indirect evidence that cannot provide the level of detail required for drawing mechanistic conclusions. We therefore set out to investigate functional and structural properties of heterologously expressed, purified and enzymatically active PDS. For this, we used a biphasic liposome-based assay system that does not require complex supplements. We also lay the basis for kinetic and mechanistic investigations which are now possible.

## Materials and Methods

### PDS-His_6_ cloning and expression

Rice *PDS* (Acc. AF049356), deprived of a stretch of nucleotides coding for the 87 amino acid transit sequence (corresponding to UniProtKB Acc. A2XDA1), was synthesized (Genescript) equipped with a 5´ *Nde*I site and 3´ *His*_*6*_ coding sequence followed by a *Hind*III site. The vector *pCRTI-His*_*6*_, used previously to express the bacterial carotene desaturase *CRTI* [12], was digested with *Nde*I and *Hind*III to remove the *CRTI-His*_*6*_—cassette that was replaced by the *PDS-His*_*6*_ coding sequence, resulting in the vector *pRice-PDSHis*_*6*_. Tuner (DE3) *E*. *coli* cells were transformed and grown in 2YT-medium under agitation at 37°C using baffled Erlenmeyer flasks. *PDSHis*_*6*_-expression was induced at OD_600_ 0.5–0.7 with 0.5 mM IPTG and the cultures kept under agitation at 15°C over night. The cultures were harvested by centrifugation and the cell pellets frozen in liquid nitrogen and stored at -80°C.

### Protein purification and analysis

*E*. *coli* cells (15 g wet weight) expressing PDS-His_6_ were suspended in 20 ml buffer A (50 mM Tris-HCl pH 8.0, 100 mM NaCl, 1 mM TCEP). After addition of DNase I, cells were disintegrated with a French Pressure Cell at 20,000 psi. Centrifugation at 19,000 x g removed cell debris and the supernatant was supplemented with 4 mM CHAPS (0.7 CMC). Talon resin (GE Healthcare) suspension was added (1 ml/5 ml supernatant) and the mixture was incubated for 30 min at 10°C under agitation, followed by centrifugation at 700 x g. The resin pellet was washed with wash buffer B (buffer A containing 500 mM NaCl and 2% glycerol), centrifuged and washed with wash buffer C (buffer B containing 10 mM imidazole). After an additional washing step with buffer A, protein elution was achieved on a column using three volumes of buffer A containing 150 mM imidazole. This was followed by dialysis against buffer D (buffer A containing 10% glycerol). This PDS-His_6_ preparation was used for enzymatic assays and for storage at -80°C, under which conditions it remained fully active for at least 6 months. For purification in the presence of norflurazon, the inhibitor was added to all buffers from an acetone stock solution to 50 μM final concentration. The protein preparation was concentrated using Vivaspin 2 concentrators (30 kDa molecular weight cut-off, Sartorius) prior to gel permeation chromatography (GPC).

Further preparative purification by GPC was used to separate different oligomeric states and for obtaining the highest purity possible for crystallization experiments. For this, we used buffer E (20 mM Tris-HCl pH 8.0, 100 mM NaCl, 100 mM imidazole in the absence or presence of 50 μM norflurazon) at a flow-rate of 1 ml min^-1^ using a Hiload 16/20 Superdex 200 prep grade or a Superose 6, 10/300 GL column (GE Healthcare) on an ÄKTA Explorer FPLC instrument (GE Healthcare) equipped with a fluorescence detector (Waters, 474 scanning fluorescence detector) to monitor the presence of FAD (Exc. = 450 nm, Em = 530 nm). PDS-His_6_ solutions were quantified using a Nanodrop photometer (Implen) using ε_280nm_ = 72,400 l mol^-1^ cm^-1^ and routinely analyzed by 12% SDS-PAGE.

Native gradient gels containing 25 mM imidazole or 50 μM norflurazon were prepared from 4% and 12% acrylamide solutions using a multiple gel caster (gel plate size 10x8 cm; Hoefer). The running buffer (200 mM glycine, 25 mM Tris) also contained 25 mM imidazole or 50 μM norflurazon.

### Crystallization and data collection

GPC-purified PDS-His_6_ was supplemented with the detergent lauryl-dimethylamineoxide (LDAO, 0.04% (w/v), Anatrace) and used for crystallization by sitting drop vapor diffusion at 20°C. 0.3 μl of protein solution (7 mg ml^–1^) were mixed with 0.3 μl of a reservoir solution containing 11% (w/v) of polyethylene glycol 2000 monomethyl ether and 0.4 M ammonium acetate buffer at pH 8.5. Crystals appeared after approximately one week as thin plates. In order to assist with experimental phase determination, the crystals were harvested into a soaking solution containing the reservoir buffer supplemented with 10 μM thiomersal (sodium 2-(ethylmercurithio)benzoate) and incubated for 5 min. To avoid ice formation, 2*R*-3*R*-butane diol was added to a final concentration of 10% (v/v) before mounting the crystals in nylon loops and flash-cooling in liquid nitrogen. Diffraction data were collected on beam line X06SA at the Swiss Light Source (Paul-Scherrer Institute, Villigen, CH). Data sets were index and integrated with XDS [26] and scaled and assessed using the AIMLESS pipeline [27]. Mercury sites were located and used for phase calculations in SHARP [28] and an initial interpretation of the resulting electron density maps was carried out with COOT [29].

### Liposome preparation and enzyme assays

Phytoene was extracted and purified from phytoene-accumulating *E*. *coli* cells, as described previously [30]. After purification, phytoene concentrations were determined photometrically in hexane solution using ε_285 nm_ = 68,125 l mol^-1^ cm^-1^.

For liposome preparation, 5 mg phosphatidylcholine were dissolved in CHCl_3_ and added to variable amounts (50 nmol under standard assays conditions) of phytoene. After vortexing, the lipid-phytoene mixture was dried under N_2_ and 1 ml liposome buffer (50 mM Tris-HCl, pH 8.0, 100 mM NaCl) was added, followed by 30 min incubation on ice. Liposomes were formed by gentle sonication. Small unilamellar vesicles were formed by a passage through a French pressure cell at 20,000 psi [30]. Phytoene concentrations in liposomes were verified using HPLC system 1 (see below).

In a final volume of 700 μl assay buffer (50 mM MES-KOH pH 6.0, 100 mM NaCl), the enzyme assay contained 25 μg of affinity-purified PDS-His_6_ (0.63 μM), 25 μM DPQ, 100 μl of liposomes (0.5 mg soybean phosphatidylcholine; PC), supplementing the assay with 7 μM phytoene. The liposomes in 100 μl were first supplemented with decyl-plastoquinone (DPQ) and vortexed, and then assay buffer was added, followed by protein. The incubation was carried out at 37°C in the dark for 10 min. The reaction was stopped by adding 1 volume of CHCl_3_/MeOH 2:1 (v/v). Quinones (Sigma-Aldrich and in part kindly provided by A. Krieger-Liszkay, Saclay, France) were added to the assays from appropriate MeOH stock solutions in volumes not exceeding 0.6 μl.

### Liposome binding assays

100 μl of PC liposomes (0.5 mg PC) were added to 50 μg PDS-His_6_ in 700 μl assay buffer (50 mM MES-KOH pH 6.0, 100 mM NaCl) and incubated for 15 min at 37°C. The assays were layered on top of a 30% sucrose cushion (in assay buffer) and centrifuged for 30 min at 110,000 x g. The liposomes were recovered from the density boundary and the bound protein was pelleted by TCA-acetone precipitation [31]. To test for ionic interactions, the recovered liposomes from the sucrose step were resuspended in assay buffer supplemented with 0.5 M KCl, incubated for 15 min and pelleted again onto the sucrose cushion. TCA-acetone precipitates were subjected to SDS-PAGE using 12% polyacrylamide gels.

### Analysis of carotenes

Carotenes were extracted from PDS-His_6_ assays with CHCl_3_/MeOH 2:1 (v/v). Extracts were supplemented with an internal standard to a concentration of either 0.3 mM tocopherol acetate (Sigma) or 1.25 μg ml^-1^ of the lipophilic metalloorganic dye VIS682A (QCR Solutions Corp). After centrifugation at 20,000 x g for 5 min, the organic phase was transferred and dried using a vacuum-concentrator (Eppendorf). Carotenoids were dissolved in 40 μl CHCl_3_ and analyzed by HPLC using a Prominence UFLC XR system equipped with a SPD-M20A PDA-detector (Shimadzu).

HPLC system 1 was used to analyze the carotene products formed. A C_30_ RP column (150 x 3 mm i.d., 5 μm; YMC) was used with the solvent system A: MeOH/*tert*-butylmethylether (TBME) 1:3 (v/v) and B: MeOH/TBME/water, 5:1:1 (v/v/v). The program started with 60% A, followed by a linear gradient to 100% A within 10 min; the final conditions were maintained for 4 min. Peaks were integrated at their individual λ_max_ and the area values corrected according to the recovery of the internal standard. A further normalization of peak areas was done according to the molar extinction coefficients of eluting carotenes. These were phytoene: ε_285 nm_ = 68,125 l mol^-1^ cm^-1^; phytofluene: ε_350 nm_ = 73,300 l mol^-1^ cm^-1^; ζ-carotene: ε_400 nm_ = 138,000 l mol^-1^ cm^-1^. Finally, amounts were determined relative to the detector response factors determined using a β-carotene standard curve.

### Chemical cross-linking and mass spectrometry

To investigate the oligomeric states of PDS-His_6_ after GPC, the protein was dialyzed against cross-linking buffer (50 mM Na_2_HPO_4_/NaH_2_PO_4_ pH 8.0, 100 mM NaCl, 5 mM TCEP) and supplemented with the membrane-permeable cross-linkers DSS, DSG, DSP and TSAT (Thermo Scientific). The reactants were added from DMSO stock solutions to achieve a concentration of 62.5 μM. After a reaction time of 5 min at room temperature, the reaction was quenched with 35 mM Tris-HCl pH 7.2. After further 15 min incubation at room temperature, the samples were analyzed by SDS-PAGE using 7.5% polyacrylamide gels.

After colloidal Coomassie staining the cross-linked PDS-His_6_, as well as the monomer band were excised from the SDS-PAGE gel and subjected to tryptic in-gel digestion. The analysis of the resulting peptides was carried out by nano-HPLC-ESI-MS/MS using an UltiMate 3000 RSLCnano/LTQ-Orbitrap XL system (Thermo Fisher Scientific) as described elsewhere [32]. LC-MS/MS data files were converted into the mzXML format using the ProteoWizard software (version 3.0.6002) [33]. Cross-linked peptides were identified using the xQuest/xProphet software (version 2.1.1) [34, 35]. Searches were performed against the amino acid sequences of the recombinant protein and of the contaminants keratin II and trypsin (UniProt accessions P35908 and P00761). Reversed sequences were created using the xdecoy tool included in the xQuest pipeline. Enzyme specificity was set to trypsin with up to two missed cleavages and a minimum peptide length of four amino acids. Oxidation of methionine and carbamidomethylation of cysteine residues were considered as variable and fixed modifications, respectively. The MS^1^ mass tolerance was set to 6 ppm, MS^2^ tolerances to 0.4 Da for cross-linker containing ions and 0.3 Da for common ions. Mass shifts were set to 96.02113 for cross-linked peptides and 114.0317 and 217.09502 for mono-linked peptides, i.e., peptides modified at a single lysine by DSG hydrolyzed or quenched by Tris, respectively. Settings for unlabeled cross-linkers were used and default values for all other parameters. xQuest combines several sub-scores into one final linear discriminant score (ld-score) for every candidate cross-linked peptide. Identifications were further filtered applying a false discovery rate of less than 5% computed using xProphet (version 2.5.1) and a minimum delta score of ≤0.95. For quantitative analysis MaxQuant (version 1.3.0.5) [36] was used with default settings, except that “match between runs” was activated. Intensities of peptide spectrum matches are based on the extracted ion currents reported in the “All peptides” result table of MaxQuant computing the sum of intensities for isotopic clusters within the MS^1^ mass tolerance of the precursor *m/z*-value with a retention time window of ± 1 min.

Cofactors (NADP(H), NAD(H), FAD, FMN) were analyzed by LC-MS using electrospray ionization in the positive ion mode, and by MS^2^-based single reaction monitoring as described previously [37]. A Surveyor HPLC system coupled to an LTQ mass spectrometer (Thermo Scientific) was used. Separation of cofactors was achieved with a 5 μm C18 reverse phase column (Hypersil Gold, Thermo Scientific) and the solvent system A (50 mM aqueous ammonium acetate in 1% formic acid) and B (1.7 mM ammonium acetate in 70% methanol acidified with 1% formic acid). The gradient was run at a flow rate of 700 μl min^-1^ from 100% A to 50% A within 10 min, with the final conditions held isocratically for 5 min. Further conditions were: capillary temperature, 350°C; source voltage 5.3 kV; capillary voltage 49 V; source current 100 μA. For cofactor identification, the following combinations of precursor and fragment ions were defined: FAD, MS^1^ *m/z* 786.2 (348.1, 439); FMN, MS^1^ *m/z* 457.1 (359.2, 439.1); NAD^+^, MS^1^ *m/z* 664.1 (524, 542.1); NADH, MS^1^ *m/z* 666.2 (348.2, 649.2); NADP^+^, MS^1^ *m/z* 744.1 (604, 622); NADPH, MS^+1^ *m/z* 746.4 (428.1, 729.1).

### Electron Microscopy

For negative staining, PDS-His_6_ samples collected from GPC (high mass peak) were adjusted to a protein concentration of 0.2–0.5 mg ml^-1^ and immediately fixed with 2.5% glutaraldehyde. Drops of 5 μl of sample were placed on glow-discharged carbon coated Ni-grids for 5 min. Samples were washed by rapidly touching the grid surface to a water drop twice, followed by rapid rinsing by touching on a drop of an aqueous solution of 2% uranyl acetate, and finally left for 30 sec on UA. After air-drying, samples were visualized in a Phillips CM10 transmission EM.

Freeze-fracture cryo-scanning electron microscopy was carried out using a phytoene-liposome suspension as isolated in the liposome binding assays including ultracentrifugation (see above). The enzymatic activity of the bound PDS-His_6_ was confirmed. The suspensions of four assays were combined, pelleted and resuspended in 40 μl incubation buffer. After addition of 30% glycerol, this was pipetted into the 50 μm cavity of two 3 mm aluminum specimen carriers. After sandwiching the two carriers, the assembly was frozen using the HPM 100 (Leica) freezer. After transfer into the Freeze Fracture System EM BAF060 (Leica) and fracturing, samples were either visualized directly in a Zeiss Auriga SEM system (-115°C, 5 kV acceleration voltage, 20 μm aperture using the inlens SE detector), or after sublimation (-105°C, 5 min) to display liposomal surfaces. The sublimated as well as the untreated samples were coated with 2.5 nm Pt/C and backed with 4 nm carbon at a gun angle of 45° and under stage rotation (40 rpm).

## Results

### Purification and Oligomeric Assembly

**Fig 2:**
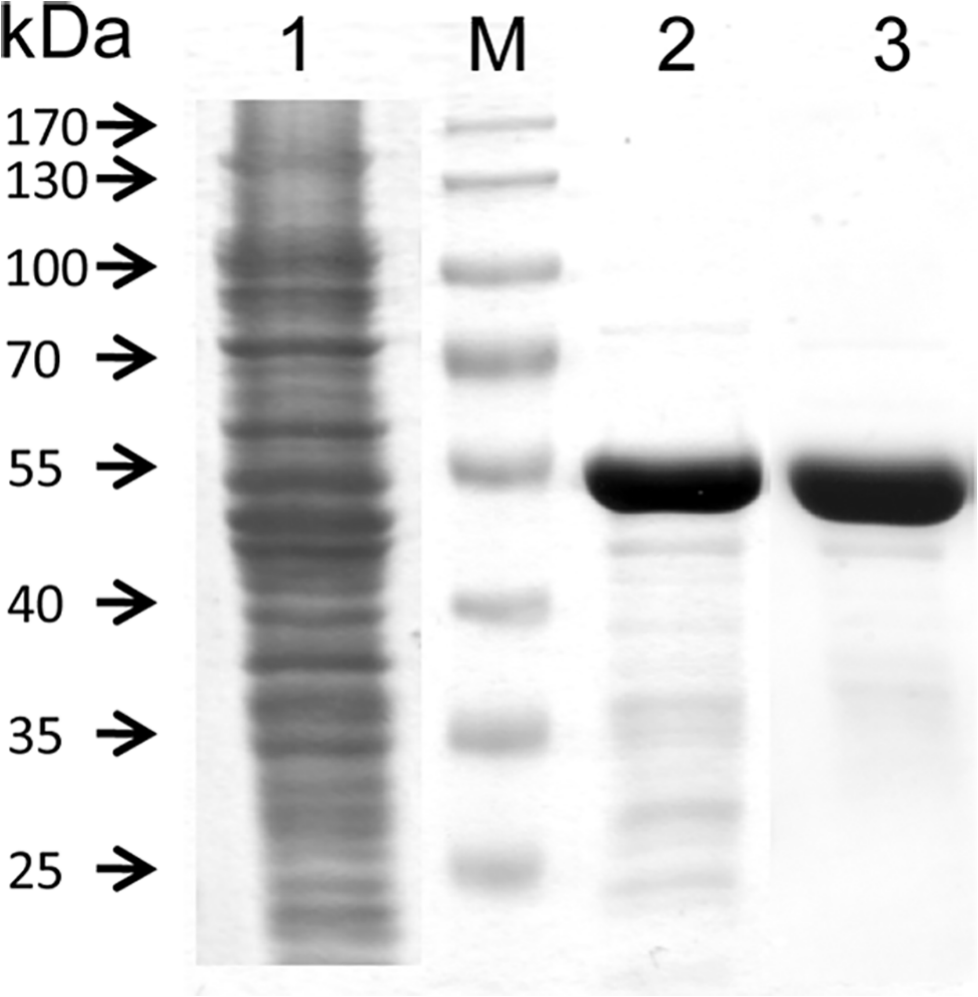
Purification of PDS-His_6_. SDS-PAGE (10%; stain Coomassie Blue) showing in lane 1, *E*. *coli* lysate after centrifugation at 18,600 x g; Lane 2, PDS-His_**6**_ after IMAC purification; lane 3, PDS-His_**6**_ after GPC purification; M, MW protein standards.

The present PDS-His_6_ purification protocol is the result of a substantial optimization required to overcome the notorious tendency of the protein for aggregation. The detergent CHAPS at concentrations well below its CMC was found to be best suited for detaching the protein from *E*. *coli* membranes while, surprisingly, no supplementation of detergents was required during all subsequent steps. The SDS-PAGE analysis depicted in Fig 2 documents a ≈ 4000-fold enrichment of PDS-His_6_ that was achieved primarily during the IMAC step (lane 2). Most of the residual contaminants were removed by GPC (lane 3), during which the presence of 150 mM imidazole was crucial. In its absence most PDS-His_6_ eluted as aggregates in the dead volume and is almost completely lost by adsorption upon ultrafiltration. On the downside, imidazole severely decreased the stability of the protein and inhibited its activity; it therefore had to be dialyzed off for both purposes. Concentrated PDS-His_6_ solutions are yellow, consistent with the presence of a flavin cofactor.

**Fig 3:**
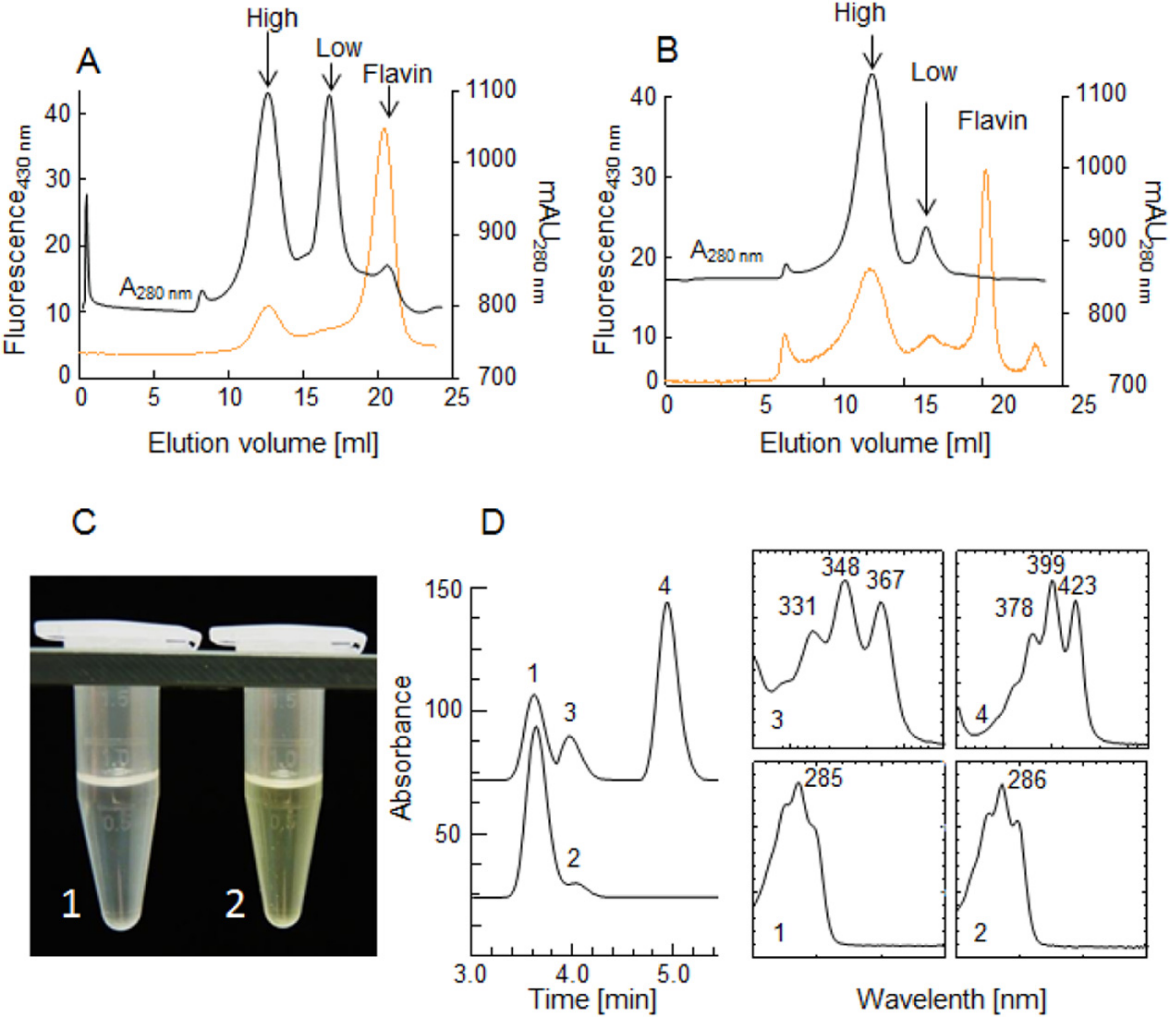
Homo-oligomers of PDS-His_6_ are enzymatically active. **A**, Upon GPC (Superose 6 10/300 GL column) of the IMAC-purified PDS-His_**6**_ only the high mass homo-oligomer (High) contains flavin as revealed by the concomitance of elution profile and florescence trace (orange; note that PDS-His_**6**_ fluorescence is quenched ≈ 5-fold compared to that of free FAD). **B**, Elution traces of PDS-His_**6**_ purified in **t**he presence of norflurazon. **C**, organic extract after an incubation of the low (1) and the high mass (2) fraction in the presence of phytoene according to standard incubation conditions as defined in the Methods section. Only the high mass population converts the colorless phytoene into the pale-yellow colored ζ-carotene. **D**, HPLC-analysis of the assays shown in C. Lower trace, no conversion with the low mass form showing the substrates 15-*cis*-phytoene (1) and traces of all-*trans*-phytoene (2). Upper trace, of incubation with high order oligomeric PDS-His_**6**_ that shows conversion of 15-*cis*-phytoene (1) into *cis*-phytofluene (3) and *cis*-ζ-carotene (4). The corresponding UV-VIS spectra are shown and numbered accordingly.

The oligomeric state of PDS-His_6_ was investigated using calibrated HiLoad Superdex 200 and Superose 6 10/300 GL columns (5–1000 kDa and 10–600 kDa separation ranges, respectively). In both cases (Fig 3A), two distinct populations of PDS-His_6_ eluted, corresponding to ≈ 56 kDa and to ≈ 450 kDa, at peak maximum. These can be assigned to the monomeric (calculated mass: 56.2 kDa) form and a population around the octameric form of the enzyme (Fig A in S1 File). Fluorescence traces recorded in parallel and photometric quantification of the flavin released after heat-denaturation are consistent with the assumption that the presumed octameric form contains approximately stoichiometric equivalents of flavin, while flavin association was much lower in the low-mass form. Accordingly, specific activities correlated with the ≈ 450 kDa elution peak, while only residual activity was detected in the low-mass peak, corresponding to ≤ 1.3% of that of the octameric form. Incubation of the high-mass form in the presence of phytoene-containing liposomes and decylplastoquinone resulted in the formation of yellowish ζ-carotene (Fig 3C). An incubation experiment showed a typical conversion rate of 6 nmol min^-1^ mg^-1^, resulting in 21% conversion of 15-*cis*-phytoene after 10 min. HPLC analysis of the extract showed the appearance of the intermediate 9, 15-di-*cis*-phytofluene, in addition to the final product 9, 15, 9´-tri-*cis*-ζ-carotene.

GPC in the presence of the herbicidal PDS inhibitor norflurazon (50 μM) indicated that the relative concentration of the flavinylated ≈ 450 kDa form is substantially increased. This is consistent with stabilization the octameric and, concomitantly, the holoenzyme forms by the inhibitor. Consequently, crystallization experiments were carried out in the presence of norflurazon.

Almost homogenously monomeric PDS-His_6_ can be obtained by GPC when the buffer is supplemented with 20 mM of the detergent CHAPS. However, this is accompanied by a complete release of the FAD cofactor (Fig B in S1 File). Attempts to reconstitute the monomeric apo-protein with FAD, thereby potentially converting it back into the oligomeric active form, were unsuccessful.

**Fig 4:**
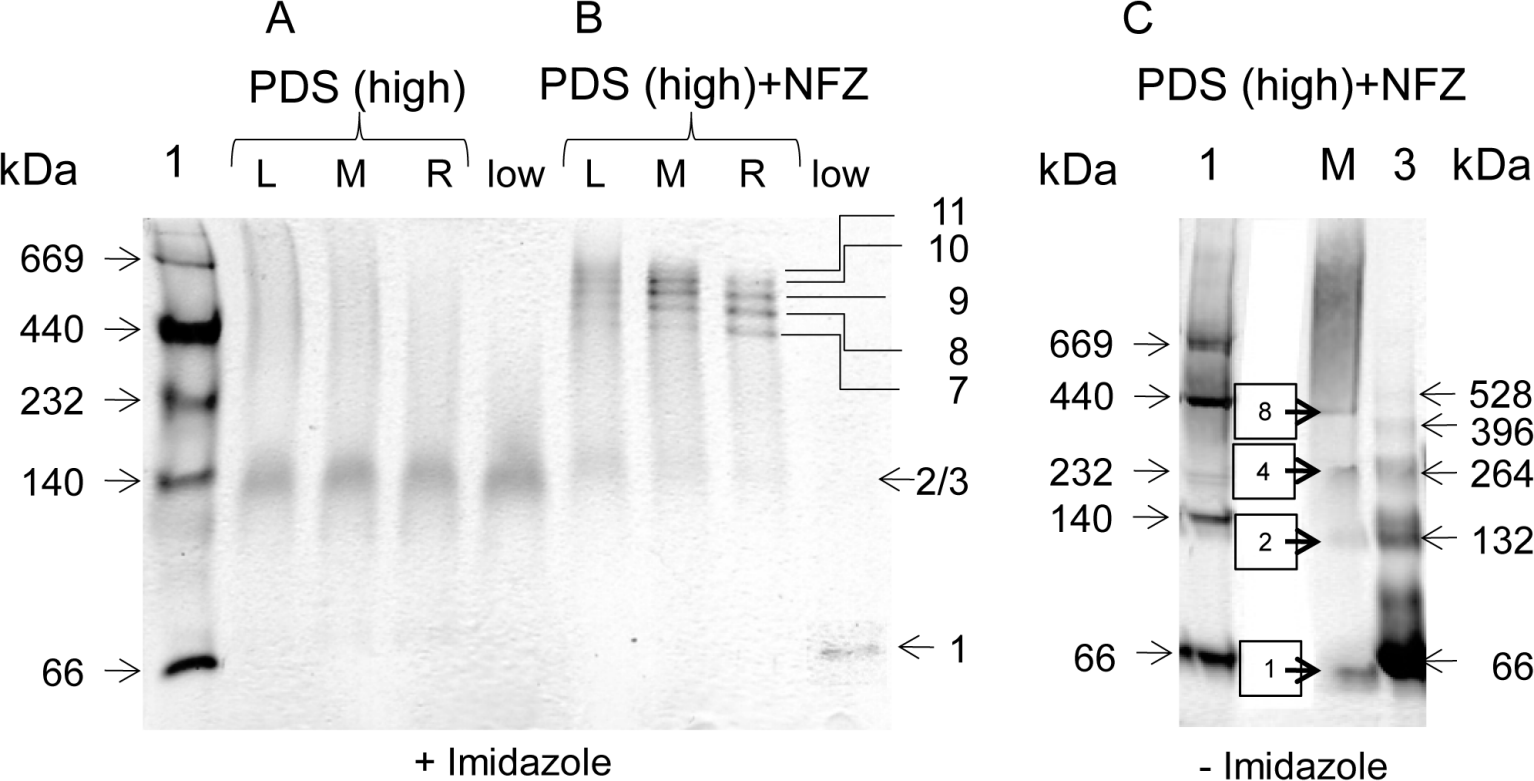
Homo-oligomers of PDS-His_6_ analyzed by native PAGE. **A**, Effect of norflurazon. The high mass oligomeric fraction from GPC (purification in the absence of norflurazon in Fig 3A) dissociates into a diffuse band representing the dimer or trimer. **B**; the high mass oligomeric fraction from GPC separation (purification in the presence of norflurazon, Fig 3B) reveals discrete bands representing the calculated oligomeric assemblies indicated. L,M,R, left, middle and right segment of the respective high mass oligomeric GPC peaks. The corresponding low mass peaks gave a diffuse dimer/trimer when the purification was done in the absence of norflurazon and the monomer in its presence (this band has been electronically contrasted, for better visibility). Gradient gels (4–12%) cast in the absence of norflurazon containing 25 mM imidazole. **C**, Effect of imidazole. PDS purified in the presence of norflurazon separated on gradient gels (as above) but cast in the absence of imidazole. Lane 1, marker proteins; M, High mass GPC peak; lane 3, homo-oligomeric forms of BSA used as a standard [53]. Calculated oligomeric states of PDS-His_**6**_ are shown as boxed.

For an analysis at higher detail than possible with GPC, PDS-His_6_ oligomers were investigated using non-denaturing gradient gels containing imidazole (25 mM). Fig 4A shows that the high-mass peaks isolated from GPC in the absence of norflurazon (comp. Fig 3A), produced a cloud of unresolved species plus a diffuse band corresponding to the mass of the dimer or trimer. In contrast, the high-mass peak isolated in the presence of the herbicide (comp. Fig 3B) yielded a pattern of discrete bands (Fig 4B). Semi-logarithmic regression analysis (Figure C in S1 File) suggests the presence of hepta- to undecamers, the difference in size corresponding to the monomer. Oligomers consisting of less than seven subunits were barely present.

To study the role of imidazole mentioned above, PDS-His_6_ isolated in the presence of norflurazon was separated by non-denaturing gradient PAGE that did not contain imidazole (Fig 4C). Most of the protein migrated as a smear corresponding to a mass >1 MDa, suggesting unspecific aggregation. Some discrete, but barely detectable bands show up at migration distances compatible with the presence of monomers, dimers, tetramers and octamers.

Non-denaturing PAGE also revealed differences within the low mass GPC peaks. While this fraction of PDS-His_6_ recovered in the absence of norflurazon (Fig 3B) revealed the same less defined dimeric or trimeric species as with the high-mass fraction, the addition of norflurazon led to a low mass fraction (Fig 3A) which showed only the monomeric species upon electrophoretic separation (Fig 4B).

### Electron microscopy of soluble and membrane-associated PDS-His_6_

**Fig 5:**
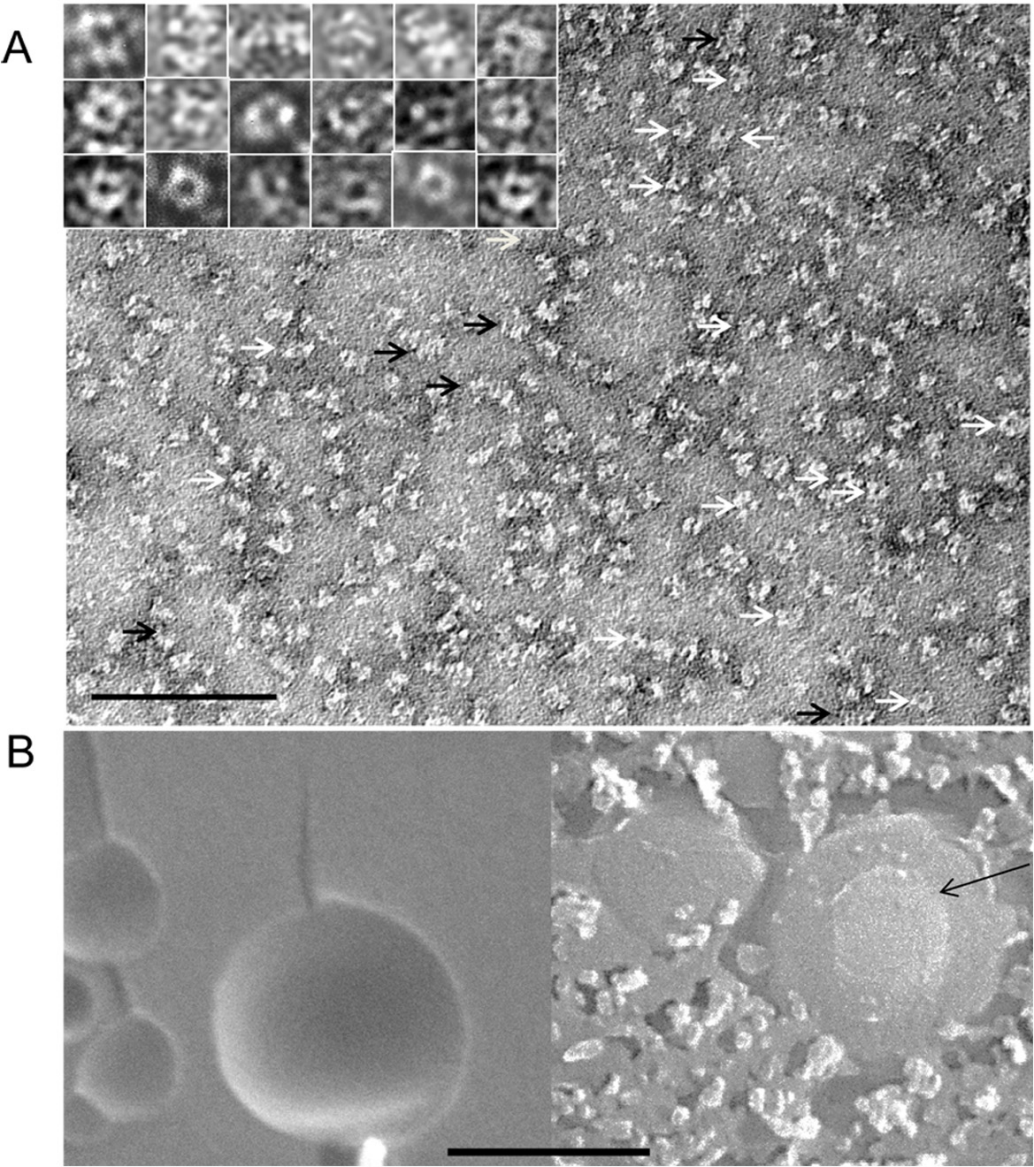
Electron microscopy of PDS-His_6_. A, negative staining. Examples of rings (white arrows) and stacks of rings (black arrows) are indicated. The inset shows examples of stacks (upper row) and rings (lower two rows) at higher magnification; each picture represents an area of 20 x 20 nm. The bar refers to the overview and represents 100 nm. **B**, Freeze-fracture scanning EM. Left, membrane fracture faces of liposomes containing bound PDS-His_**6**_ showing the absence of transmembrane particles. Right, membrane surfaces exposed after sublimation. The arrow points to the surface/fracture face boundary. Particles of homogenous size are seen on the surface. Bar represents 200 nm.

Negative staining of the higher-order oligomeric species purified in the presence of norflurazon revealed a distribution of particles, among which two recurrent patterns were observed consisting of rings (white arrows) and stacks (black arrows; Fig 5A). Rings appeared mostly four- membered, with a diameter of 11.8 ± 1.3 nm (n = 40). Apparently, such rings assemble into stacked tubular structures (seen in a side view) with a similar diameter of 10.7 ± 1.2 nm (n = 30). Stacks were variable in length ranging from 15–30 nm. Where discernible, the number of stack layers suggest a monomer height of ca. 4–5 nm. Additional structures that could not be integrated into these two categories are probably caused by insufficient focus and/or the different angles under which the structures are viewed. We interpret the stacked rings to cause the higher-order oligomers (≈ 450 kDa at GPC peak maximum) seen on native gels and upon GPC. Such large, three-dimensional arrays may represent artifacts caused by the absence of membranes, while rings may represent the functional units, bearing in mind that only higher-order homo-oligomers are enzymatically active.

To elucidate the situation at liposomal membranes, to which PDS-His_6_ binds spontaneously (see below), freeze-fracture electron microscopy was carried out (Fig 5B). Enzymatically active PDS-His_6_-membrane associates were used for this purpose. The fracture faces were free of particles, indicating that PDS-His_6_ does not dip deeply into membranes, probably representing a monotopic membrane protein. Sublimation uncovered membrane surfaces on which particles were seen that were 14.5 ± 1.9 (n = 30) nm in diameter. Considering the addition of the ca. 2 nm caused by the Pt/C-coating, this distribution fits well with the size of the ring structures and is not in favor of a functional relevance of the higher order associates at membrane surfaces.

**Fig 6:**
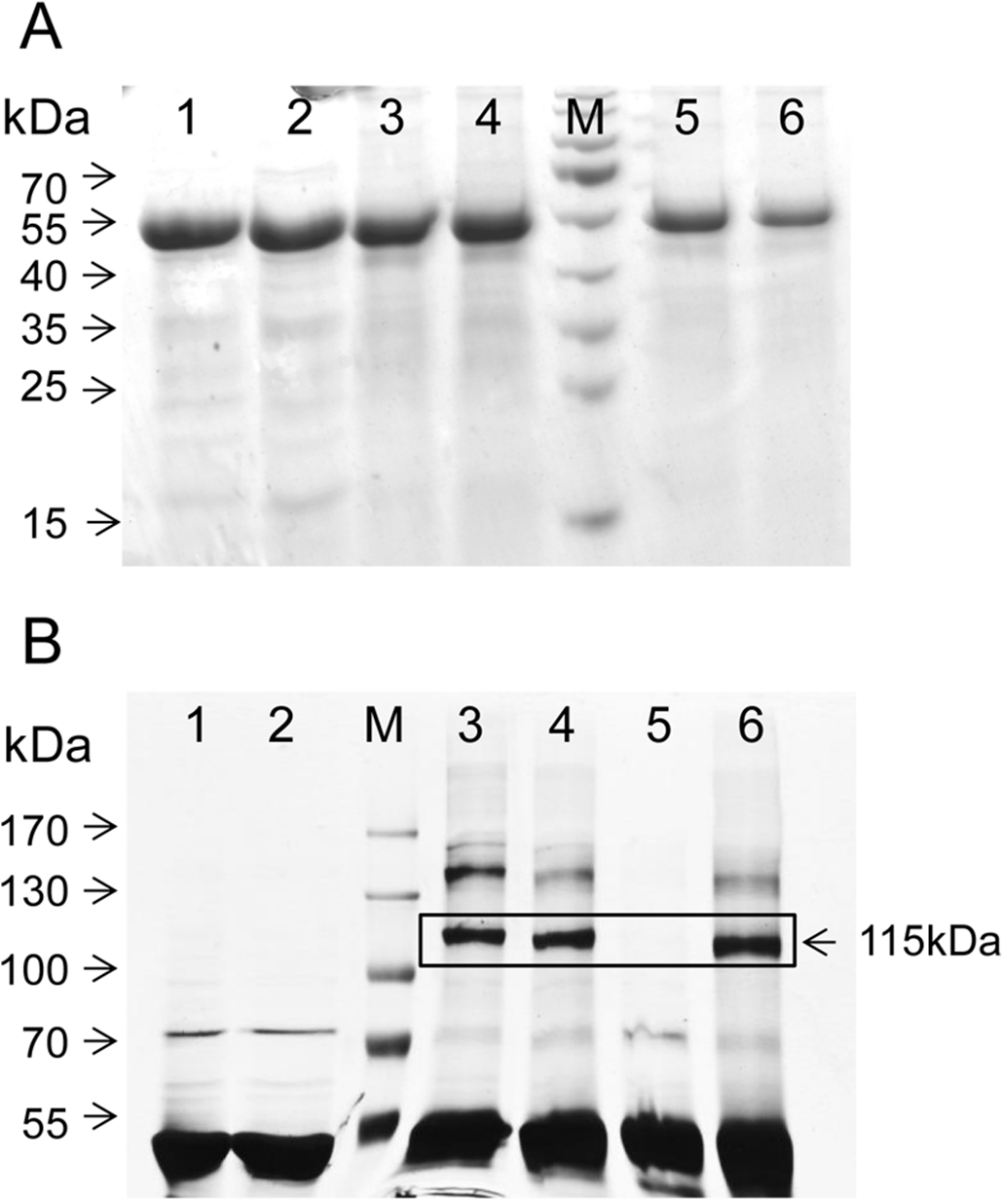
Membrane association and chemical cross-linking of PDS-His_6_. **A**, SDS-PAGE (12%, Coomassie Blue-stained) analysis of liposomal binding assays. Lanes 1 and 2; 50% of the PDS-His_**6**_ amount added to the liposomal suspension (25 μg). Lanes 3 and 4 represent 100% of the liposome-bound PDS-His_**6**_, after centrifugation onto a 30% sucrose cushion. Equal band strengths observed thus indicate ca. 50% protein binding. These liposomes were washed either with incubation buffer (lane 5) or 0.5 M KCl in incubation buffer (lane 6) and recollected by ultracentrifugation. 100% of this material was applied. The sample duplication represents independent experiments. **B**, SDS-PAGE (12%, silver stained) of cross-linked PDS-His_**6**_ collected from the high mass oligomeric GPC peak obtained in the absence of norflurazon (as shown in Fig 3A). Lanes 1, 2, untreated PDS; M, marker proteins. The chemical cross linkers used were DSS (lane 3), DSG (lane 4), DSP (lane 5) and TSAT (lane 6). Dimeric reaction products were predominantly formed (boxed).

PDS-His_6_, although purified as a soluble protein, must be able to interact with membranes since the carotene substrate provided in liposomal membranes resides within the hydrophobic core [38]. In fact, the protein binds spontaneously to liposomal membranes (Fig 6A). Ultracentrifugation of liposomes onto a 30% sucrose cushion after addition of PDS-His_6_ showed ≈ 50% protein recovery in the liposome band. High-salt (0.5 M KCl) treatment of these isolated liposomes followed by additional centrifugation revealed that the interaction is apparently hydrophobic, since the protein was retained. The somewhat weaker bands observed are probably due to certain losses of liposomes caused by the repeated centrifugation and collection.

### Chemical crosslinking and mass spectrometry

To study the oligomeric assembly of PDS-His_6_ in greater detail, chemical cross-linking was carried out, using the high-mass fraction isolated in the absence of norflurazon (as shown in Fig 3A). SDS-PAGE analysis of PDS-His_6_ treated with the amine-reactive, non-cleavable NHS esters DSS, DSG and TSAT revealed silver-stained protein bands at approx. 115 kDa (Fig 6B, lanes 3, 4 and 6), corresponding to PDS-His_6_ dimers (calculated mass 112.4 kDa). Further, weaker bands observed at approx. 150 kDa may represent PDS-His_6_ trimers. A substantial proportion remained unlinked and dissociated into monomers during denaturing PAGE. Coomassie-stained gel bands of PDS-His_6_ dimer cross-linked by DSG and the monomer band were excised and the tryptic digests were analyzed by mass spectrometry in order to identify vicinal peptides. Candidate cross-linked products were identified by their monoisotopic masses matching the predicted combinations of two tryptic peptides derived from PDS-His_6_ concatenated by the cross-linking reagent. Matches were further validated by interpretation of the respective MS^2^ spectra. Comparative analysis of intensities of quasi-molecular ions of cross-linked peptides in MS^1^ spectra from PDS-His_6_ dimer and monomer indicated that several crosslinks were specifically formed in the dimer (matches 1 to 7, Table 1), pointing towards intermolecular contact sites, which is further supported by the identity of the cross-linked peptides 4 and 6 (Table 1). Together with the molecular mass of the cross-linked protein, these data suggest that the enzyme forms a homodimer and the higher order oligomers may reflect its multiple forms. The cross-linked peptides 8 and 9 likely represent intramolecular connections as the same species were identified for the cross-linked PDS-His_6_ monomer (Table 1).

**Table 1:** Cross-linked peptides of phytoene desaturase identified by mass spectrometry.

	Mass (Da)	Peptide A Sequence	Site	Peptide B Sequence	Site	ld-score	Intensity	Band
**1**	2910.5422	SPIEGFYLAGDYTKQK	K534	ILKYHVVK	K495	33.40	5.9E7	D
**2**	2465.2924	LCAQSVVEDYKMLSR	K560	AKILK	K492	33.37	1.2E7	D
**3**	2760.5600	YLADAGHKPILLEAR	K129	ILKYHVVK	K495	32.10	3.5E7	D
**4**	3727.8100	SPIEGFYLAGDYTKQK	K534	SPIEGFYLAGDYTKQK	K534	31.69	2.9E7	D
**5**	3577.8375	SPIEGFYLAGDYTKQK	K534	YLADAGHKPILLEAR	K129	26.86	8.1E6	D
**6**	1128.6721	KLEK	K394	KLEK	K394	26.12	1.0E6	D
**7**	3441.6899	LCAQSVVEDYKM*LSR	K560	LFPDEIAADQSKAK	K490	24.16	4.2E6	D
**8**	3133.6000	LFPDEIAADQSKAK	K490	LKNTYDHLLFSR	K415	24.01	1.6E8	M,D
**9**	3261.6906	LFPDEIAADQSKAK	K490	KLKNTYDHLLFSR	K413	22.48	5.3E7	M,D

Sites of cross-linking are underlined. The numbering refers to UniProtKB Acc.No. A2XDA1, phytoene desaturase *Oryza sativa subsp*. *indica*. M^*^, oxidized methionine; ld-score, combined score for identification; D, dimer band (MW ≈ 55 kDa); M, monomer band (MW ≈ 115 kDa).

### Preliminary structural analysis

**Fig 7:**
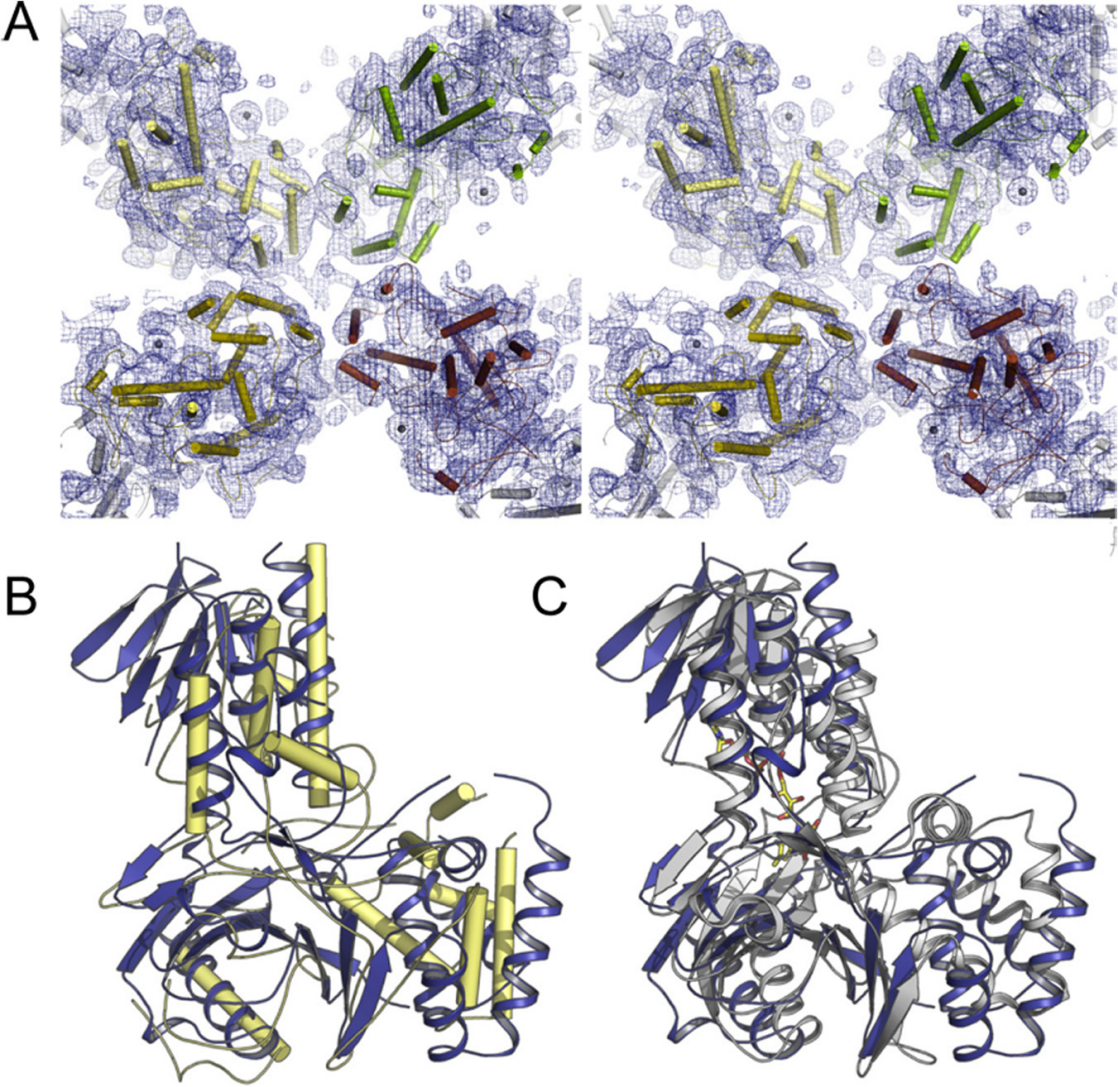
Preliminary structural analysis of PDS-His_6_ by X-ray crystallography. **A**, Stereo representation of a representative section of the experimental electron density map as a blue mesh, with initial α-helical models shown as cylinders. **B**, Superposition of a preliminary PDS-model shown as yellow cylinders with the structure of CRTI (PDB-ID: 4DGK) in blue, displaying the high similarity in the overall structure of the two enzymes. **C**; Cartoon representation of a superposition of CRTI in blue with an oxidoreductase from *Methanosarcina mazei* (PDB-ID: 3KA7) in grey and its FAD cofactor in yellow.

Diffracting single yellow colored crystals of PDS-His_6_ were obtained by sitting-drop vapor diffusion, and the mercuric compound thiomersal was successfully employed to address the crystallographic phase problem. In the best diffraction data sets available at present, the anomalous signal of mercury extended to a maximum resolution of 7.5 Å, and phase information calculated on this basis was iteratively extended to yield an experimental electron density map at 6 Å resolution (Fig 7A). Data collection statistics are summarized in Table 2. While this was not yet of sufficient quality to build an atomic model for the enzyme, the map showed unambiguous solvent boundaries and continuous stretches of electron density that likely represent α-helices. This led to a first low-resolution model for PDS-His_6_ with a kinked two-domain architecture that underlines a homotetrameric arrangement of the protein in the crystals (Fig 7A). The homotetramer is generated through a crystallographic two-fold axis. Even at the present resolution, the model obtained for PDS-His_6_ shows clear similarities to the structure of CRTI (Fig 7B).

**Table 2:** Data collection statistics.

	Peak dataset	Low remote dataset
**Space group**		*P* 4_1_2_1_2
**Wavelength [Å]**	1.00684	1.01126
**Unit cell axes [Å]**		177.9, 177.9, 254.5
**Unit cell angles [°]**		90.0, 90.0, 90.0
**Resolution [Å]**	67.4–4.4 (4.7–4.4)	67.5–5.0 (5.48–5.0)
***R*_merge_**	0.353 (1.68)	0.327 (2.16)
***R*_p.i.m._**	0.095 (0.336)	0.065 (0.426)
**Mean *I*/σ(*I*)**	10.2 (2.8)	11.1 (2.2)
**Completeness [%]**	99.9 (99.9)	99.9 (99.8)
**Anomalous completeness [%]**	99.9 (99.9)	99.9 (99.8)
**Multiplicity**	26.3 (26.5)	26.0 (26.3)
**CC_1/2_**	0.998 (0.935)	0.999 (0.900)
**DelAnom CC_1/2_**	0.251 (0.031)	-

Values in parentheses represent the highest resolution shell.

### Cofactors and the effect of norflurazon

Based on the MS data (M^+1^ *m/z* 786.2, fragment ions: *m/z* 439.2 and *m/z* 348.1), the UPLC elution profiles and UV-VIS spectra, the yellow cofactor of PDS-His_6_ can unambiguously be identified as FAD (Figures A-C in S2 File). The redox-cofactors FMN(H_2_) and NAD(P)(H) were absent.

**Fig 8:**
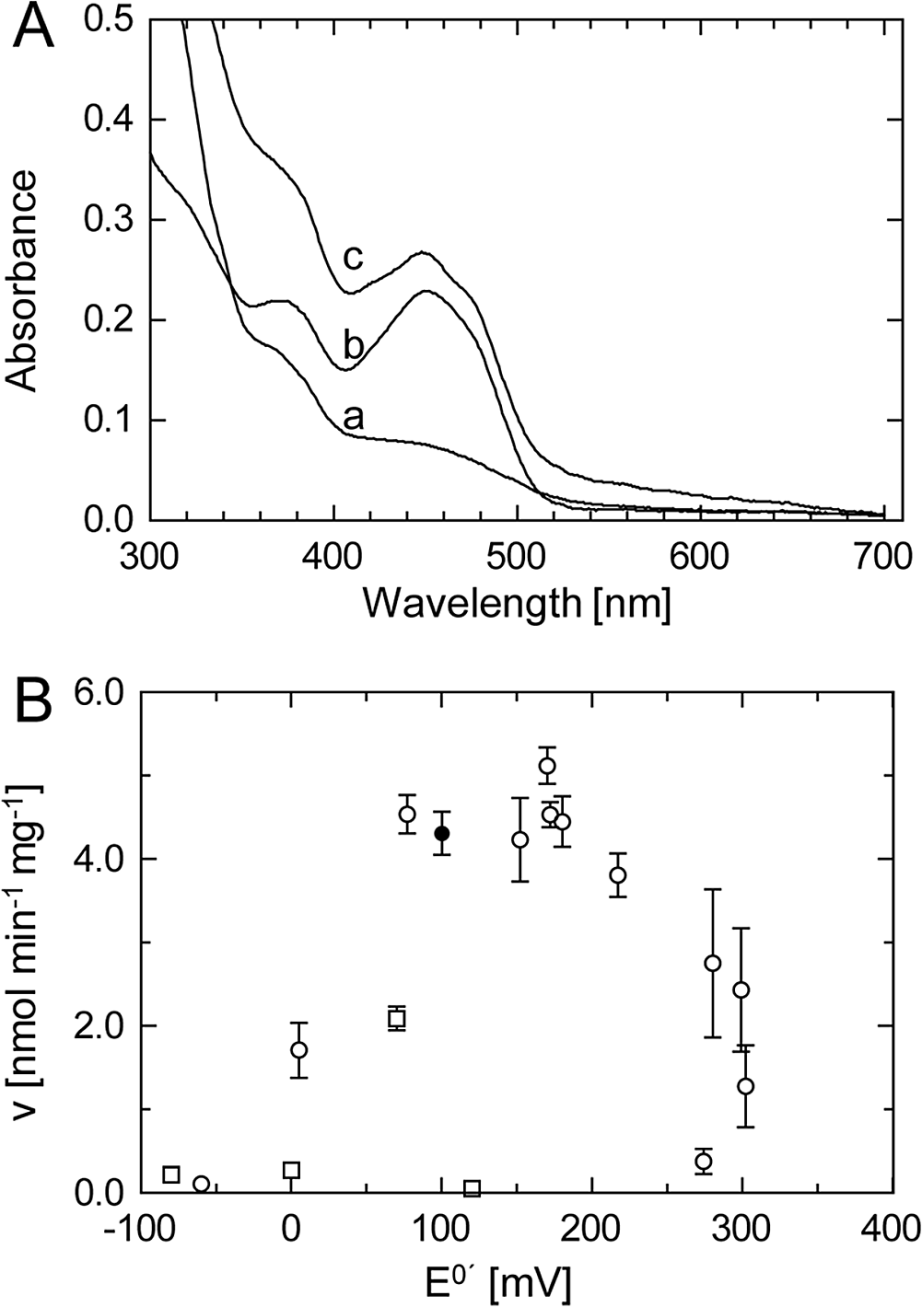
Spectral properties of PDS and roles of quinones and of norflurazon. **A** Trace; UV-VIS spectra of PDS-His_**6**_ that was purified in the presence of norflurazon. The low absorbance in the 450 nm region and the high one in the 310 nm range indicates the presence of enzyme-bound reduced flavin (spectra recorded after 2h at RT in an oxygen atmosphere). Trace b, spectra of the same sample upon heat-denaturation of the protein (5 min 100°C). Trace c; spectrum of PDS upon addition of 200 μM DPQ (recorded after 20 min). **B**, Dependence of the rate of ζ-carotene formation on the E^o’^and structure of the quinone electron acceptor. The structures of the naphtoquinones (☐) and benzoquinones (○) used are given in S3 File. Decyl-plastoquinone (100 mV) is highlighted (●).

The presence of the herbicide norflurazon (50 μM) in all buffers used during purification led—in addition to the stabilizing effect discussed above—to the isolation of the reduced, colorless PDS-His_6_ protein. The reduced form was stable for over 2 h in ambient oxygen atmosphere (Fig 8A, trace a). Heat denaturation led to rapid oxidation upon FAD-release and appearance of yellow color (trace b). We deduce from this observation that the PDS-His_6_-bound FAD is reduced by an unknown donor in *E*.*coli* and that the reduced colorless form is stabilized by association with norflurazon. Interestingly, rapid reoxidation can also be achieved with excess decyl-plastoquinone (DPQ; trace c), indicating that the quinone and norflurazon compete at the FAD site.

Quinones are co-substrates of PDS, and barely any activity can be obtained in their absence. Therefore, the structural determinants for this effect were investigated (Fig 8B; see S3 File for structures). Benzoquinones were strongly preferred over naphthoquinones, which is in favor of the participation of plastoquinone over phylloquinone *in vivo*. There was relaxed specificity for the various benzoquinones used. Optimal catalytic effectiveness was achieved with those ranging between E^0´^+100 mV and +200 mV. Among these was decyl-plastoquinone, alkylated at C10 to mimic prenylation. It was therefore used in all further activity experiments. On the other hand, the plastoquinone head group 2,3-dimethyl-*p*-benzoquinone was similarly effective indicating a minor importance of the hydrophobic tail for the molecular interaction with PDS-His_6_.

**Fig 9:**
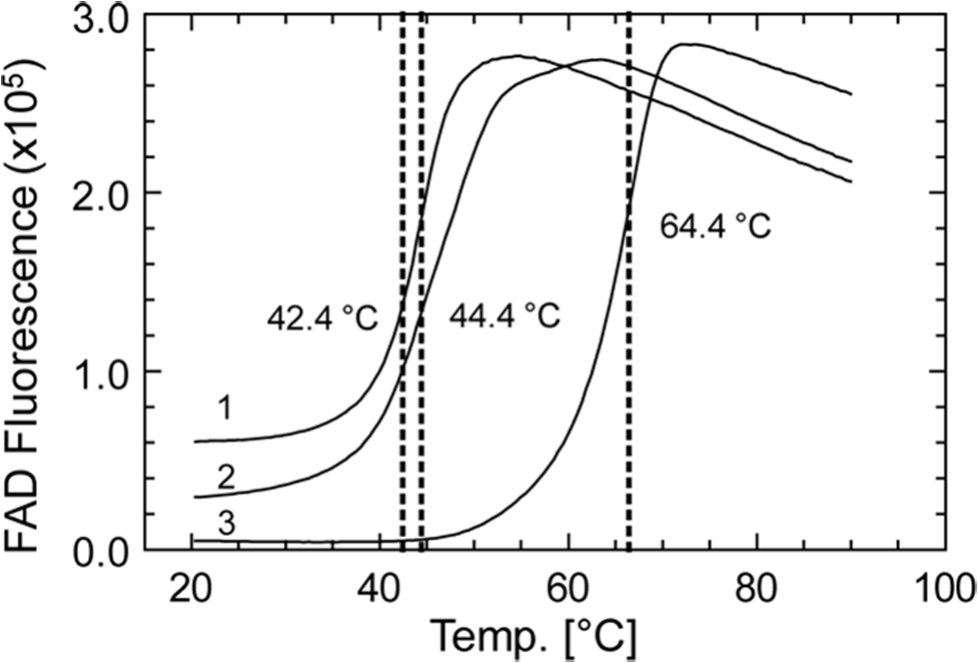
Effect of the redox state of FAD and of norfluorazon on the stability of PDS-His_6_. Thermo-FAD measurements were carried out in a RealTime-PCR instrument using the SYBR-Green Channel (Exc = 488 nm; Em = 520 nm). Trace 1; PDS-His_**6**_ isolated in the absence of norflurazon containing FAD_**ox**_. Trace 2; PDS-His_**6**_ isolated in the absence of norflurazon containing FAD_**ox**_ after addition of 50 μM norflurazon, Trace 3; PDS isolated in the presence of 50 μM norflurazon containing FAD_**red**_.

Alterations of FAD fluorescence can be used to monitor (un)folding processes of flavoproteins in response to temperature [39]. Fig 9 shows such studies with PDS-His_6_, purified in the absence of norflurazon therefore containing FAD_ox_ (Fig 6A, curve 1). The increase in fluorescence shows an inflection point at 44°C, reflecting denaturation. Addition of norflurazon to the oxidized protein modifies this response towards a more biphasic behavior accompanied and by a small increase in thermostability (curve 2). A drastic increase in thermostability of ≈ 22°C is observed with PDS-His_6_ that was isolated in the continuous presence of norflurazon and thus contained reduced FAD (curve 3). This can point towards a substantial structural change of the enzyme in response to the redox state of the flavin.

## Discussion

The results presented add PDS-His_6_ to the group of carotenoid biosynthesis enzymes that show high enzymatic activity *in vitro* using phosphatidyl-choline liposomes not requiring radiolabeled substrates for activity monitoring. This group includes the bacterial phytoene desaturase CRTI [12], the bacterial (CRTY) and plant (LCYe) lycopene cyclases [11, 38] and the plastohydroquinone:oxygen oxidoreductase PTOX [21] that links the activity of PDS to the redox state of the plastoquinone pool. Carotenes are thought to diffuse within the hydrophobic core of membranes [39]. PDS-His_6_, behaving soluble during purification, has a high binding affinity to phospholipids (Fig 5B and Fig 6A) yielding a productive micro-topology allowing the exchanging of substrate and product with the lipid phase.

Although phospholipids are quantitatively dominated by galactolipids in plastid membranes, negatively charged membrane surfaces may be required for membrane association. The absence of particles from membrane fracture faces (Fig 5B) suggests that PDS-His_6_ associates membrane-peripherally. PDS-His_6_ does not exhibit hydrophobic sequence regions that might be assumed to span membranes; therefore it may dip monotopically into membranes in order to gain substrate access. A similar topology has been postulated for CRTI [30].

Examples for 3D-characterized monotopic membrane proteins utilizing long-chain isoprenoids as substrates are represented by the retinal forming oxygenase ACO [40] and the structurally related retinoid isomerase RPE65 [41]. Furthermore, squalene-hopene cyclase [42] attains this topology and violaxanthin de-epoxidase, capable in binding reversibly to the thylakoid membranes, interacts in a similar way [43].

Monotopic membrane proteins can form oligomeric assemblies that shield their hydrophobic surfaces from water and behave as soluble units [44]. In fact, PDS-His_6_ forms high-order oligomers in solution with an apparent mass ≈ 450 kDa (n = ca. 8) at GPC peak maximum, however, considerably higher and also lower numbers of monomers are present (Fig 3A). These homo-oligomers are unstable upon non-denaturing PAGE producing a cloud of unresolved oligomeric species; only a faint band representing dimers or trimers can be detected (Fig 4A). High-order oligomers are stabilized upon binding of norflurazon as witnessed by GPC (Fig 3B) and upon non-denaturing PAGE (Fig 4B), now showing discrete oligomers of n = 7–11 and higher, while lower order associates are practically absent. It is likely that such assemblies represent dissociated forms of even higher associates that disaggregate upon electrophoresis. The presence of imidazole prevents catastrophic aggregation into very large >1 MDa aggregates that are prone to precipitation and adsorption on ultrafiltration membrane surfaces (Fig 4C).

The presence of norflurazon during the entire purification also leads to the isolation of colorless PDS-His_6_ that contains reduced FAD (Fig 8A). The Thermo–FAD experiments (Fig 9) indicate that norfluorazon has an only marginal effect on the stability of oxidized PDS (curves 1, 2). However it induces a substantial stabilization when bound to PDS-His_6_ containing reduced FAD. The difference in thermostability indicates that the protein exists in substantially different conformations depending on the redox state of the cofactor. This and the homo-oligomeric state of the active protein (Fig 3C and 3D) may form the basis of the pronounced cooperativity of the system noted in kinetic studies (to be published).

PDS-His_6_ is a flavoprotein containing FAD. Spectroscopic observations with isolated PDS suggested the presence of a flavin cofactor [2]; evidence was then presented that the enzyme is flavinylated after plastid import in a Hsp70-bound form, concomitant with membrane binding [45]. A substantial influence of NAD^+^ and NADP^+^ and ineffectiveness of FAD described by others for a PDS from *Synechococcus* [1] may be due to secondary effects caused by the complexity of the system used, i.e. the addition of crude fungal extracts to the purified enzyme.

Two scenarios can be considered to explain the observed stability of PDS containing FAD_red_ and norflurazon towards dioxygen. Norflurazon might form an adduct with the reduced flavin. Such a species may exhibit drastically reduced dioxygen reactivity. More likely, the binding of the inhibitor might shield the active center from dioxygen, as has been observed e.g. with reduced medium-chain acyl-CoA dehydrogenase [46]. The effect of norflurazon is reversible. Rapid reoxidation is observed with the electron acceptor decyl-plastoquinone (Fig 8, trace c). Whether norflurazon and quinones compete for the same FAD oxidation site remains to be demonstrated.

Significantly, very low catalytic activity is observed in the presence of dioxygen but in the absence of norflurazon and quinones. This is in marked contrast to the case of the bacterial phytoene desaturase CRTI, for which the reoxidation of FAD_red_ by O_2_ is a catalytically necessary step. Thus, while CRTI is an oxidase, PDS-His_6_ appears as an obligatory phytoene: benzoquinone oxidoreductase (Fig 8B). Benzoquinones with an E^0´^ of around +150 mV lead to the highest activity. This coincides with the earlier observations that chromoplast membranes at an ambient redox potential of ca. +100–200 mV (attributable to the redox state of plastoquinone), are optimal for phytoene desaturation [19]. The observation that Arabidopsis mutants blocked in the biosynthesis of plastoquinone cannot desaturate phytoene [18] strongly corroborates the inability of dioxygen to reoxidize PDS and that no alternative electron acceptor is available *in vivo*.

Consequently, PDS is redox-controlled *in vivo* being dependent on the redox state of the plastoquinone pool. This relates to the activity of the photosynthetic redox chain in chloroplasts and to the activity of the plastid terminal oxidase (PTOX, a hydroquinone:oxygen oxidoreductase) in non-green plastids [20]. CRTI, being not redox-controlled and quinone-independent under aerobic conditions, may be causal for its effectiveness in Golden Rice [47] where the enzyme is not rate-limiting, even at very low expression levels [48].

Clearly, PDS-His_6_ oligomerization is a structural prerequisite for functionality, since only the high molecular mass fraction collected from GPC is enzymatically active (Fig 3C and 3D). This goes along with only the higher-order oligomers retaining FAD while the low molecular mass forms are prone to lose this cofactor. The complete dissociation of PDS oligomers into apoprotein monomers by the detergent CHAPS suggests that oligomerization and the concomitant maintenance of the bound FAD relies on hydrophobic interactions and that monomers might be ineffective in binding FAD. This leads to the question on the minimal oligomeric unit required for enzymatically activity.

This question is difficult to address, experimentally. We have resorted to freeze-fracture electron microscopy allowing to distinguish the size of a tetramer and the larger tubular structures. Clearly, no such large >15 nm structures were seen. Instead, the particles seen on liposomal surfaces, but absent from facture faces, were evenly size-distributed with a diameter matching the one of the tetrameric ring. This speaks against the involvement of the higher order oligomers as well as of the monomer and suggests the tetrameric ring structures as a catalytically active unit at membrane surfaces. In fact, PDS examined by Blue Native-PAGE from several plant species revealed a membrane-associated ca. 350 kDa PDS species and a stromal population of ca. 660 kDa [49]. While the latter may be due to complex formation with the plastid chaperone system [45], the former might reflect this tetrameric species. It needs to be noted that homo-oligomerization, membrane binding and the PQ redox state may not be the only determinants of PDS activity. Evidence is accumulating that differences in the sub-plastid topology and the formation of protein heterocomplexes may impact pathway fluxes and thus the enzymatic activity of its constituent members as reviewed in [50].

Chemical cross-linking showed the preferential formation of a dimeric species and mass spectrometry revealed interacting peptide sequences which will be helpful in assessing the biological relevance of the three-dimensional crystal structure, once resolved. So far, the 6 Å electron density maps obtained at the current state of structural analysis of PDS-His_6_ quite clearly point towards an association in the form of tetramers in which dimers may represent the functional unit. The diameter of the tetramer of ca. 140 nm leaving a central space matches well with the tetrameric ring-like structures visualized by EM. The overall arrangement strengthens the assumption that a highly extended substrate such as 15-*cis*-phytoene cannot be accommodated by a single monomer, but is rather processed by a dimer, with one protomer working on each end of the symmetrical phytoene substrate simultaneously, or consecutively. Our initial analysis of the layout of a PDS monomer shows clear similarity to the GR_2_ family of flavoproteins including CRTI, and a structural superposition (Fig 7B and 7C) highlights the relation between three members of this family.

In a conceivable interpretation of the available data, PDS-His_6_ is thus assumed to consist of a membrane-bound tetramer that is composed of dimers as the functional unit. A dimer as the functional unit was also postulated with the bacterial desaturase CRTI, based on structural data and *in silico* docking experiments [30]. However, the similarities extend beyond the level of oligomeric assembly. Although CRTI from *Pantoea ananatis* and OsPDS share only 22% amino acid sequence similarity and 11% identity, much of which is attributed to the FAD-binding Rossmann fold common to both, and although there are clear differences in catalysis, like the differential roles of oxygen, quinones, stereochemistry and the number of double bond introduced, the overall protein folds are quite similar. This places OsPDS and CRTI into a structural context with monoamine oxidases and protophorphyrinogen oxidases as suggested previously based on sequence comparisons of extended Rossman fold domains [51] and homology modeling [52]. This implies that that the two desaturases have evolved divergently or convergently resulting in two different approaches towards achieving similar catalytic goals. A more detailed discussion on this topic will rely on refined structural information and mechanistic insights.

## Supporting Information

S1 File Molecular mass determination of PDS-His_6_ oligomersGPC of PDS-His_6_ isolated in the absence of norflurazon on a calibrated HiLoad Superdex 200 column. The values for K_AV_ and the derived apparent molecular masses are indicated (**Figure A)**. GPC of PDS-His_6_ in the presence of 20 mM CHAPS leads to complete disaggregation into monomeric subunits and concomitant release of the flavin cofactor. Orange, FAD fluorescence (**Figure B**). Molecular mass estimation of PDS-His_6_ oligomers resolved on non-denaturing gradient gels (**Figure C**). The calculated apparent molecular masses are indicated, revealing an incremental difference approximately matching the mass of the monomer.
(TIF)

S2 File Analysis of the flavin cofactor bound to PDS-His_6_. Quasi-molecular ion representing the M^+1^ of FAD (MW = 785.5 Da) (**Figure A**). The two expected MS^2^ FAD fragment ions (**Figure B**). UV-Vis spectrum of the FAD released from PDS by heat denaturation and centrifugation (**Figure C**).
(TIF)

S3 File Structures of the naphtoquinones and benzoquinones used (in ascending order of their E0´): (1) Menaquinone (-80 mV); (2) 2,5-dihydroxy-benzoquinone (-60 mV); (3) menadione (0 mV); (4) 2,3,5,6-tetramethyl-p-benzoquinone (duroquinone; 5 mV); (5) naphtoquinone (70 mV); (6) 2,6-dimethyl-p-benzoquinone (77 mV); (7) decyl-plastoquinone (100 mV); (8) 1,2-naphthoquinone-4-sulfonate (120 mV); (9) phenyl-p-benzoquinone (152 mV); (10) 2,3-dimethoxy-5-methyl-p-benzoquinone (170 mV); (11) 2,3-dimethyl-p-benzoquinone (172 mV); (12) 2,5-dimethyl-p-benzoquinone (180 mV); (13) 2,6-dichlorphenol-indophenol-Na; DCPIP, 217 mV); (14) 3,5-di-tert-butyl-1,2-p-benzoquinone (274 mV); (15) p-benzoquinone (280 mV);) (16) 2,5-dichloro-p-benzoquinone (299 mV); (17) 2,6-dichloro-p-benzoquinone (302 mV). (TIF)
