# Supplementary figures and images for: Phytoene Desaturase from Oryza sativa: Oligomeric Assembly, Membrane Association and Preliminary 3D-Analysis
Source: PLoS One. 2015 Jul 6;10(7):e0131717. doi: 10.1371/journal.pone.0131717 (PMC4492965; doi:10.1371/journal.pone.0131717)

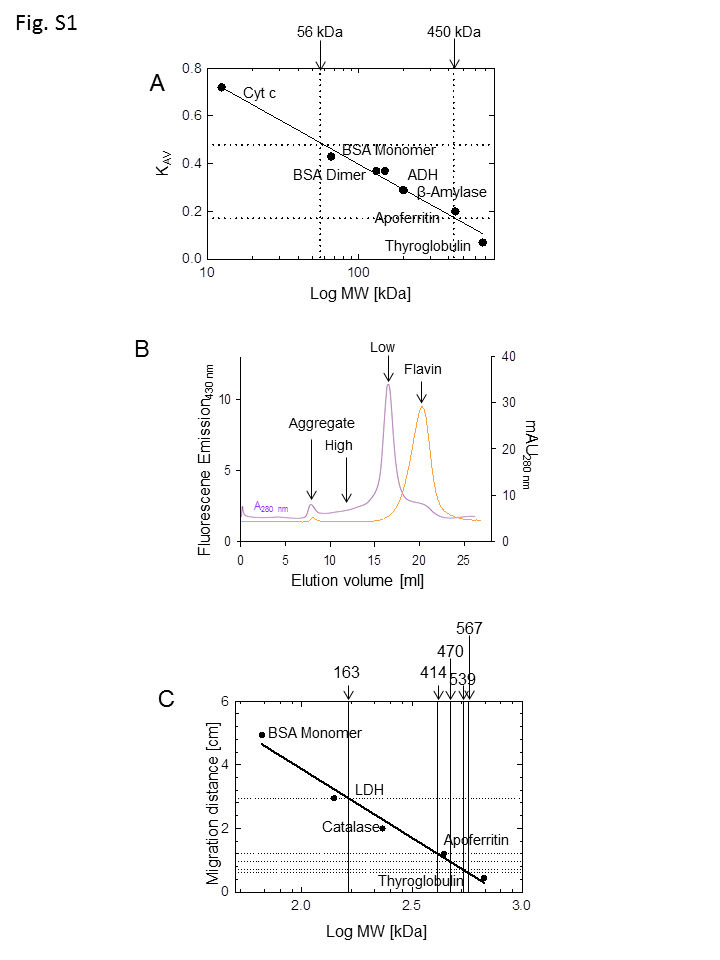

Supplement: S1 File — The values for KAV and the derived apparent molecular masses are indicated (Figure A). GPC of PDS-His6 in the presence of 20 mM CHAPS leads to complete disaggregation into monomeric subunits and concomitant release of the flavin cofactor. Orange, FAD fluorescence (Figure B). Molecular mass estimation of PDS-His6 oligomers resolved on non-denaturing gradient gels (Figure C). The calculated apparent molecular masses are indicated, revealing an incremental difference approximately matching the mass of the monomer. (TIF) [file pone.0131717.s001.TIF]

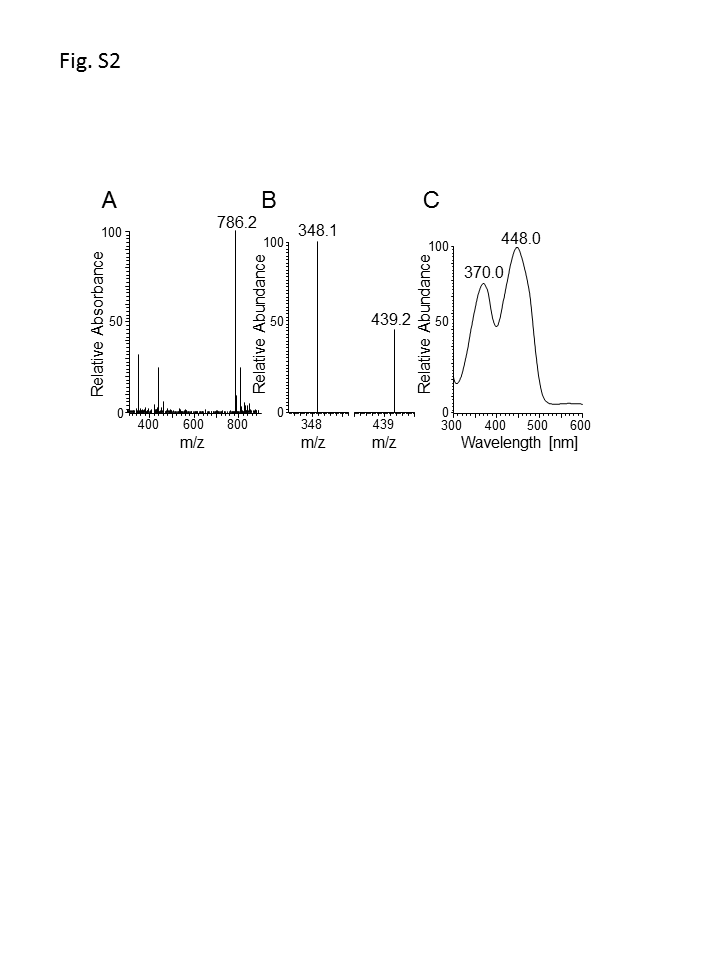

Supplement: S2 File — Quasi-molecular ion representing the M+1 of FAD (MW = 785.5 Da) (Figure A). The two expected MS2 FAD fragment ions (Figure B). UV-Vis spectrum of the FAD released from PDS by heat denaturation and centrifugation (Figure C). (TIF) [file pone.0131717.s002.TIF]

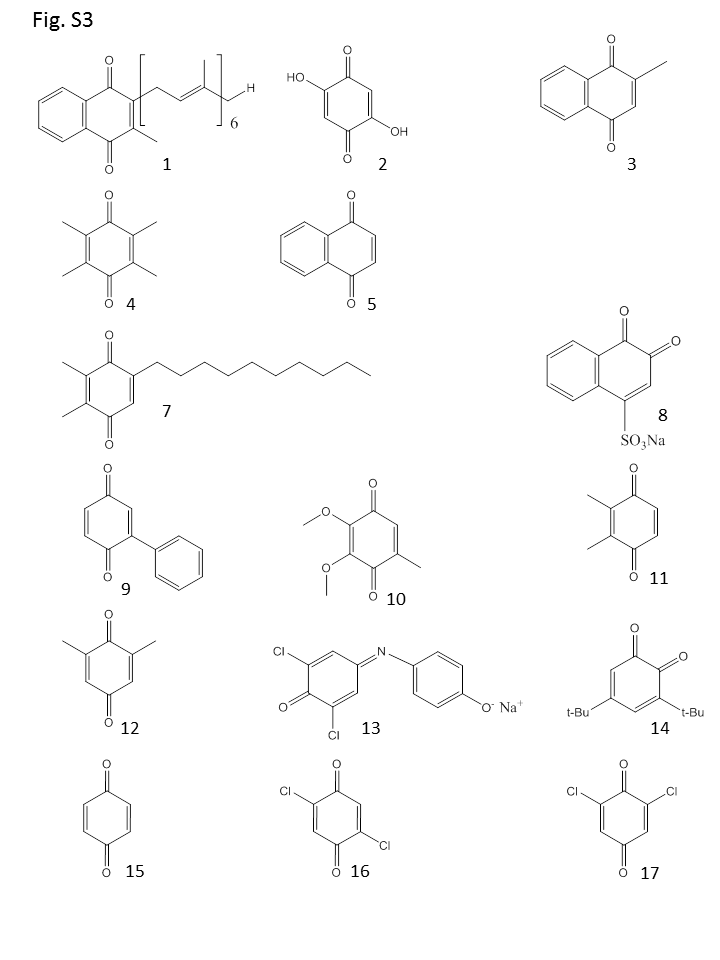

Supplement: S3 File — (TIF) [file pone.0131717.s003.TIF]
